# Transcriptional landscape of pulmonary lymphatic endothelial cells during fetal gestation

**DOI:** 10.1371/journal.pone.0216795

**Published:** 2019-05-13

**Authors:** Timothy A. Norman, Adam C. Gower, Felicia Chen, Alan Fine

**Affiliations:** 1 Pulmonary Center, Boston University School of Medicine, Boston, Massachusetts, United States of America; 2 Pathology & Laboratory Medicine, Boston University School of Medicine, Boston, Massachusetts, United States of America; 3 Clinical and Translational Science Institute, Boston University School of Medicine, Boston, Massachusetts, United States of America; 4 Boston Veteran’s Hospital, West Roxbury, Massachusetts, United States of America; Cincinnati Children's Hospital Medical Center, UNITED STATES

## Abstract

The genetic programs responsible for pulmonary lymphatic maturation prior to birth are not known. To address this gap in knowledge, we developed a novel cell sorting strategy to collect fetal pulmonary lymphatic endothelial cells (PLECs) for global transcriptional profiling. We identified PLECs based on their unique cell surface immunophenotype (CD31+/Vegfr3+/Lyve1+/Pdpn+) and isolated them from murine lungs during late gestation (E16.5, E17.5, E18.5). Gene expression profiling was performed using whole-genome microarrays, and 1,281 genes were significantly differentially expressed with respect to time (FDR *q* < 0.05) and grouped into six clusters. Two clusters containing a total of 493 genes strongly upregulated at E18.5 were significantly enriched in genes with functional annotations corresponding to innate immune response, positive regulation of angiogenesis, complement & coagulation cascade, ECM/cell-adhesion, and lipid metabolism. Gene Set Enrichment Analysis identified several pathways coordinately upregulated during late gestation, the strongest of which was the type-I IFN-α/β signaling pathway. Upregulation of canonical interferon target genes was confirmed by qRT-PCR and *in situ* hybridization in E18.5 PLECs. We also identified transcriptional events consistent with a prenatal PLEC maturation program. This PLEC-specific program included individual genes (*Ch25h*, *Itpkc*, *Pcdhac2* and *S1pr3*) as well as a set of chemokines and genes containing an NF-κB binding site in their promoter. Overall, this work reveals transcriptional insights into the genes, signaling pathways and biological processes associated with pulmonary lymphatic maturation in the fetal lung.

## Introduction

The earliest LEC progenitors express Prox1, a required lymphatic transcription factor, in a distinct subset of anterior cardinal venous endothelial cells at ~E9.5 in the mouse [1–4] though several groups have suggested additional non-venous origins [5–11]. Vegfr3 activation by Vegf-C induces Prox1+ LEC progenitors to emerge from cardinal and intersomitic veins to form the jugular lymph sacs and subsequently migrate into peripheral tissues [12–16]. In the early fetal lung, LECs migrate as single cells into the lung parenchyma beginning around E10.5-E11.5 prior to lumen formation [17].

During late gestation, the lymphatic vasculature undergoes dynamic structural changes as they gain the capacity to transport cells and macromolecules. This provides evidence that pulmonary lymphatics undergo functional maturation prenatally [18–20]. This process may be part of a broader, generalized paradigm whereby cellular maturation in the late fetal lung precedes the transition to air breathing. Examples include alveolar type-II cell surfactant biosynthesis, macrophage-mediated surfactant catabolism, alveolar type I cell flattening, as well as vascular and interstitial remodeling [21–24]. Accordingly, the complex pathology of the premature lung likely reflects maturational defects in multiple cell types and signaling events.

For survival after birth, a mature pulmonary lymphatic system is necessary for proper immune surveillance and lung fluid homeostasis. In this context, two groups have shown that fetal lymphatic ablation results in lung fluid retention and respiratory distress at birth [17, 19] though the effect of lymphatic ablation on immune function was not examined. Notably, alterations in lymphatic structure and function have been implicated in lung diseases that afflict neonates and young children such as bronchopulmonary dysplasia, pulmonary lymphangiectasia, congenital chylothorax and asthma [25].

Despite the fundamental importance of lymphatics for optimal lung function and their implication in respiratory pathologies, little is known regarding the transcriptional mechanisms associated with pulmonary lymphatic maturation prior to birth. To address this gap in knowledge, we established a cell sorting method to isolate CD45-/Epcam-/CD31+/Vegfr3+/Lyve1+/Pdpn+ primary PLECs from fetal murine lungs for transcriptional profiling using microarrays.

Here, we identified differentially expressed genes and pathways involved in vascular development, complement and coagulation cascades, lipid biosynthesis and metabolism and the innate immune response. The type I interferon (IFN-I) signaling pathway was the gene set with the greatest coordinate upregulation from E16.5 to E18.5, which was confirmed by qRT-PCR and *in situ* hybridization. Furthermore, NF-kB target genes, chemokines and additional genes were upregulated specifically in late-gestation PLECs. Together, these data provide novel insights into the transcriptional mechanisms, biological processes and key signaling events associated with lymphatic maturation in the fetal lung, a critical event for air breathing and postnatal survival.

## Materials and methods

### Mice and tissue processing

Our protocol was approved under Boston University IACUC Protocol# 14419. Timed pregnant mice (C57BL/6NCrl) were ordered from Charles River Labs and cared for in the BU Animal Science Center on a 12-hour light:dark cycle with access to food and water *ad libitum*. Timed pregnant dams and fetal offspring were ethically euthanized under deep isoflurane anesthesia followed by harvesting of lung tissues from E16.5, E17.5 and E18.5 mice for PLEC isolation.

On gestational days E16.5 and E18.5, fetuses from anesthetized pregnant mice were removed from the gravid uterus prior to dissection of the lungs and heart *en bloc* in ice-cold Hank’s Buffered Saline solution (HBSS). Tissues were fixed in fresh 4% paraformaldehyde (Ted Pella) at 4°C overnight. Specimens were washed in PBS (pH 7.4), dehydrated through 50%, 70%, 85%, 100% ethanols and 100% xylene (30 min/each). Tissues were then incubated at 60°C in 1:1 xylene:paraffin wax (1 hr), 100% paraffin (2 hr) and then embedded in paraffin molds at 4°C. E16.5 and E18.5 paraffin sections were mounted on Superfrost *Plus* glass slides (Fisher Scientific).

### Immunostaining and confocal imaging

Prior to immunostaining, sections were de-paraffinized, rehydrated and processed for antigen retrieval via the microwave method in citrate buffer (H-3300, Vector Labs), washed in PBS and blocked for 1 hr in 0.1%Tween-20 in PBS and 5% normal donkey serum. Antibodies used were: goat anti-Prox1 (R&D, AF2727), rat anti-Vegfr3 (Novus, AFL4), rat anti-Lyve1 (Novus, ALY7), rabbit anti-CD31 (Abcam, ab28364) and Syrian hamster anti-Pdpn (DSHB, 8.1.1). Alexa Fluor conjugated secondary antibodies used were: donkey anti-goat AlexaFluor647, donkey anti-rat AlexaFluor488, donkey anti-rat AlexaFluor594, donkey anti-rabbit AlexaFluor-594 (all from Invitrogen), and rabbit anti-Syrian hamster AlexaFluor-488 (Jackson Immunologics). Maximum intensity projections of 8–10 μm z-stack images were acquired on a Zeiss LSM 710 NLO confocal microscope using the smart setup tool in sequential scanning mode.

### *In situ* hybridization

E16.5 and E18.5 fetal lungs were fixed in 4% paraformaldehyde overnight, processed, and paraffin embedded according to manufacturer’s recommendations. Antigen retrieval was carried out on 8 um lung sections (n = 3 mice/time point) via the microwave method. All reagents for peroxidase quenching, protease digestion, washing, hybridization and transcript detection for *Ifit1* (ACDBio, cat# 500071) were purchased from the manufacturer and used according to the recommendations for the RNAscope method (ACDBio). In order to identify PLECs, sections were concomitantly blocked and immunostained as mentioned with goat anti-Vegfr3 (AF2727, R&D Systems).

### Fluorescence activated cell sorting and RNA extraction

Timed pregnant C57BL/6N dams (Charles River Labs) at embryonic days E16.5, E17.5, E18.5 were euthanized by cervical dislocation under isoflurane anesthesia. Samples (n = 3) for each time point consisted of pooled embryonic lungs from two litters. Embryonic lungs were dissected in ice-cold HBSS, minced and enzymatically digested at 37°C for 1 hr at 200 rpm in 5 mL HBSS containing 5 ug/ml collagenase II (Worthington), 2.5 mM CaCl_2_, 10 U/ml DNase I followed by gentle trituration every 15 mins. After 1 hr, digests were inactivated with addition of two volumes of FACS buffer (2% FBS/5 mM EDTA in HBSS without Ca^2+^ or Mg^2+^), centrifuged at 500g x 7 mins at 4°C, resuspended in 5 mL FACS buffer, passed through 70 μm and 40 μm cell strainers, and then centrifuged at 500 g x 7 mins at 4°C. The whole lung cell pellets were gently disrupted in 2 mL Hybri-Max RBC lysis buffer (Sigma-Aldrich) for 1 min, diluted with 10 mL FACS buffer and centrifuged at 500 g x 7 mins at 4°C. Pellets were resuspended in 1 mL FACS buffer, blocked with CD16/32 antibodies (1:100) for 10 mins, and then immunostained with the following antibodies: CD45-eFluor450 (1:500), Epcam-eFluor450 (1:50), Pdpn/8.1.1-PE (1:100), Lyve1-eFluor660 (1:100), CD31-PerCP710 (1:100) (all from EBioscience), and Vegfr3-AF488 (1:50) (Novus, AFL4). Dead cells were discriminated using 1:250 dilution of LIVE/DEAD Aqua viability dye (ThermoFisher). Immunostaining was performed for 30 min on ice in the dark before washing with 1 mL FACS buffer, centrifugation and resuspension in 500 ul FACS buffer. Cell sorting experiments were performed on a FACS Aria SORP and analyzed with FlowJo v10.2. All sorted cell populations were collected directly into RNA lysis buffer. RNA was extracted using the miRNEasy kit (Qiagen) cDNA was generated using Superscript III reverse transcriptase (ThermoFisher). Samples with an RNA Integrity Number (RIN) ≥ 7.5 were retained in the analysis.

### Microarray analysis of fetal pulmonary lymphatic endothelial cells

Mouse Gene 2.0 ST CEL files were normalized to produce log2-transformed gene-level expression values using the implementation of the Robust Multiarray Average (RMA) in the affy R package (version 1.36.1) and an Entrez Gene-specific probeset mapping (version 17.0.0) from the Molecular and Behavioral Neuroscience Institute at the University of Michigan (the "Brainarray" group). Array quality was assessed by computing Relative Log Expression (RLE) and Normalized Unscaled Standard Error (NUSE) using the affyPLM R package (version 1.34.0); all samples had median RLE and NUSE values below 0.1 and 1.05, respectively. Principal Component Analysis (PCA) was performed using the *prcomp* R function with expression values *z*-normalized across all samples (set to a mean of zero and a standard deviation of one). Differential expression was assessed using the moderated (empirical Bayesian) ANOVA and *t* test implemented in the limma R package (version 3.14.4), i.e., creating simple linear models with lmFit, followed by empirical Bayesian adjustment with eBayes. Correction for multiple hypothesis testing was accomplished using the Benjamini-Hochberg false discovery rate (FDR). All microarray analyses were performed using the R environment for statistical computing (version 2.15.1). The raw and processed gene expression data have been deposited in the Gene Expression Omnibus (GEO), Series GSE121079.

### Functional annotation

Mouse Entrez Gene identifiers from each cluster were analyzed using the Database for Annotation, Visualization and Integrated Discovery (DAVID, v6.8) with the GOTERM_BP_DIRECT collection to identify Gene Ontology (GO) biological process terms with significant overrepresentation.

### Gene set enrichment analysis

The Entrez Gene identifiers of the human homologs of all mouse Entrez Genes present in the probeset mapping described above (identified using HomoloGene, version 68) were ranked according to the *t* statistic computed between the E16.5 and E18.5 time points. The ranked list was limited to only those human genes that correspond 1:1 with a mouse homolog, and used to perform pre-ranked GSEA (version 2.2.1, default parameters with random seed 1234) using the Entrez Gene versions of the Biocarta, KEGG, and Reactome (c2 CP) motif (c3) and GO (c5) gene sets obtained from the Molecular Signatures Database (MSigDB), v5.0.

### Comparative microarray analysis of whole fetal lung, GSE35485

Mouse Gene 1.0 ST CEL files were obtained from the Gene Expression Omnibus (GEO), Series GSE35485, which profiled gene expression in whole lung during perinatal maturation. Analysis was limited to the samples from the E16.5, E17.5, and E18.5 timepoints in C57BL/6 animals. Normalization to Entrez-Gene-specific expression values, statistical analysis, and GSEA were performed in the same manner and with the same software versions and parameters as described above for the PLEC microarrays.

### qRT-PCR

Independent PLEC RNA samples were generated to validate microarray gene expression changes and to assess interferon pathway gene expression. Taqman qRT-PCR primers used to validate microarray expression data are as follows: *Prox1*, Mm00435969_m1; *Vegfr3*, Mm01292604_m1; *Lyve1*, Mm00475056_m1; *Emcn*, Mm00497495_m1; *Gbp4*, Mm00657752_m1; *Ifit1*, Mm00515153_m1; *Irf7*, Mm00516788_m1; *Socs3*, Mm01249143_g1; *Stat1*, Mm00439518_m1; *Stat2*, Mm00490887_g1; *Mcm5*, Mm01243763_g1; Actb, #4351315 (Thermo-Fisher Scientific).

### Statistical analysis

All statistical analyses utilized a one- or two-tailed Student’s *t* test as indicated in each figure legend. P values less than 0.05 were deemed significant.

## Results

### Immunophenotypic analysis and isolation of pulmonary lymphatic endothelium

**Fig 1:**
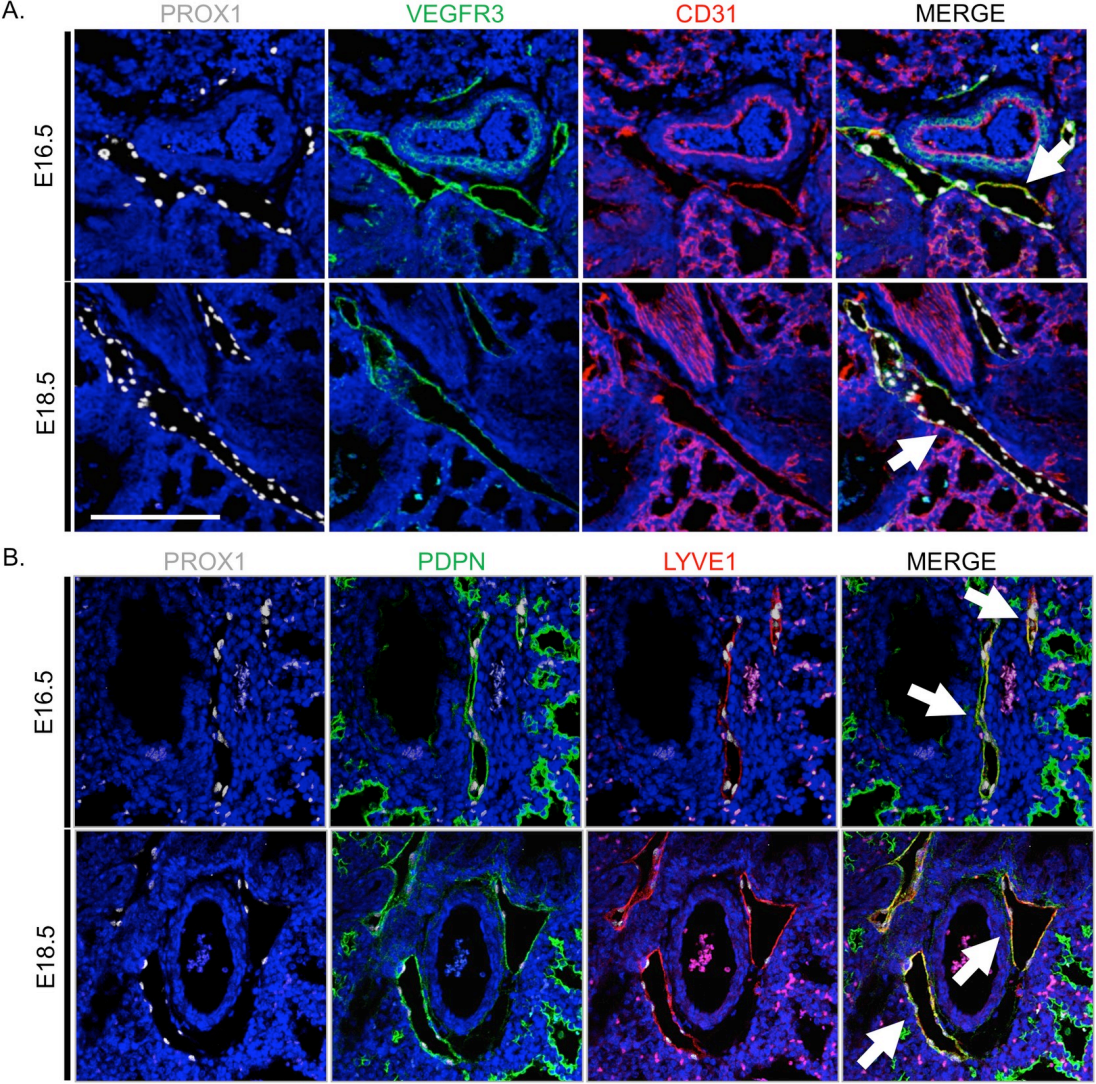
Immunophenotypic characterization of pulmonary lymphatic endothelium at E16.5 and E18.5. (A) Prox1 (*white nuclei*) in fetal lungs colocalized with Vegfr3 (*green*) and the pan-endothelial marker CD31 (*red*). (B) Prox1 (*white*) also colocalized with Pdpn (*green*) and Lyve1 (*red*) at E16.5 and E18.5. Colocalization of Vegfr3, CD31, Pdpn and Lyve1 staining was observed only in pulmonary lymphatic endothelium as indicated by white arrows (*scale bar*, *100 um)*.

To identify genes, gene networks and signaling pathways associated with LEC maturation in the fetal lung, we developed a cell sorting strategy to isolate pulmonary LECs (PLECs) for broad-based transcriptomic profiling. This approach is based on the identification of a unique panel of LEC surface markers that immuno-colocalized with Prox1, the essential transcription factor required for lymphatic development. We found that CD31, Vegfr3, Pdpn, and Lyve1 immuno-colocalization with Prox1 was only detected in lymphatic vessels in the E16.5 and E18.5 lung (Fig 1).

**Fig 2:**
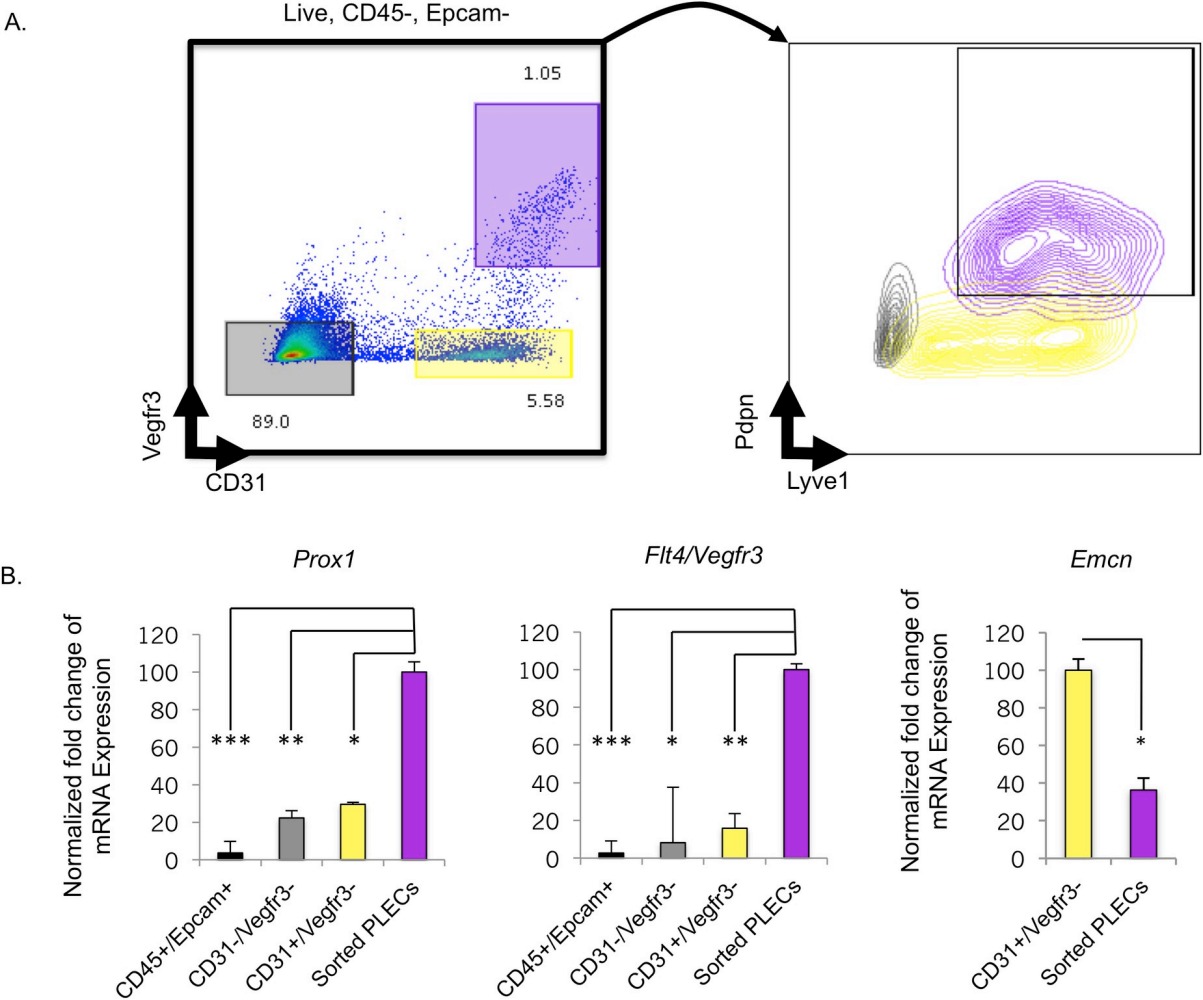
Isolation and relative purity of sorted CD45-/Epcam-/ CD31+/Vegfr3+/Lyve1+/Pdpn+ PLECs. (A, *left*) Cell sorting algorithm depicting three major cell populations; CD31-/Vegfr3- (grey), CD31+/Vegfr3- (yellow), CD31+/Vegfr3+ (purple) observed after excluding CD45+, Epcam+ and dead cells (E18.5 shown). (A, *right*) Expression of Lyve1 and Pdpn in each of the three cell fractions. (B) qRT-PCR measurements of relative *Prox1*, *Flt4/Vegfr3*, and *Emcn* expression in each sorted lung fraction. Statistical significance was determined using a two-tailed Student’s *t* test (***p<0.*001*, **p<0.*01*, *p<0.*05*; n = 3 per cell fraction).

**Fig 3:**
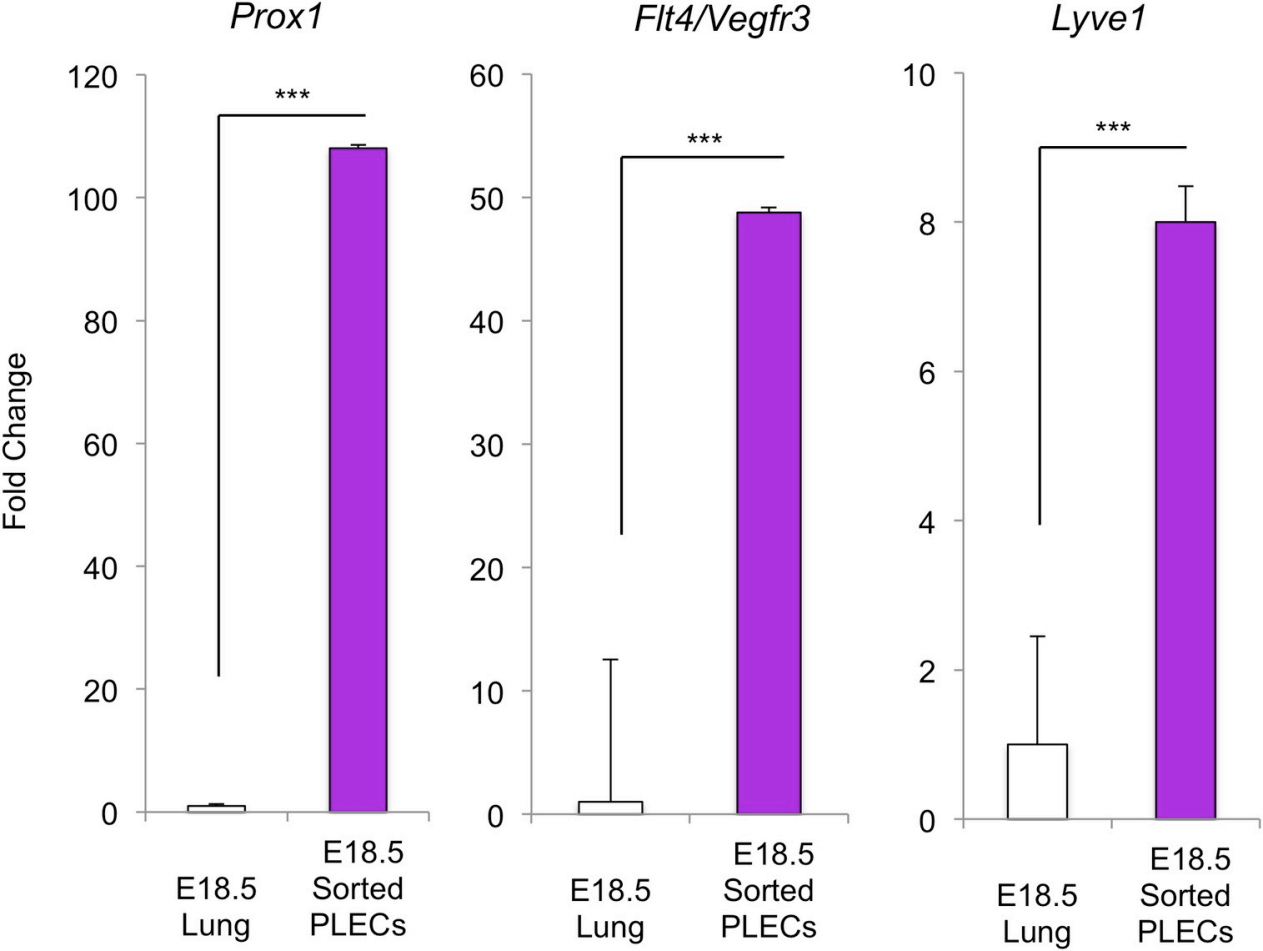
Enrichment of lymphatic markers in sorted PLECs. qRT-PCR measurement of the lymphatic markers, *Prox1*, *Flt4/Vegfr3*, and *Lyve1* in sorted E18.5 PLECs (n = 4) versus E18.5 whole lung (n = 3). Statistical significance was determined using an unpaired two-tailed Student’s *t* test (***p<0.*001*).

To isolate LECs by flow cytometry, we analyzed cell fractions in collagenase digested lung preparations after staining with the four lymphatic directed antibodies that co-localized with Prox1 (Fig 1). Following exclusion of doublets, dead cells, CD45+ hematopoietic cells and Epcam+ lung epithelial cells, we identified three major cell populations based on CD31 and Vegfr3 expression (Fig 2A) during cell sorting. The CD31+/Vegfr3+ double positive fraction consistently accounted for ~1–2% of total viable cells of which 85–95% also expressed the lymphatic markers Lyve1 and Pdpn (Figs 2A and 3, *right panels*). A subset of CD31+/Vegfr3- cells expressed Lyve1 but not Pdpn, consistent with a venous endothelial phenotype [26] (Fig 2A, yellow cluster,). Importantly, dual expression of both Pdpn and Lyve1 was only observed in the CD31+/Vegfr3+ gate (Fig 2A, purple cluster). Collectively, these data indicate that CD31+/Vegfr3+/Pdpn+/Lyve1+ cells are highly enriched in PLECs.

**Fig 4:**
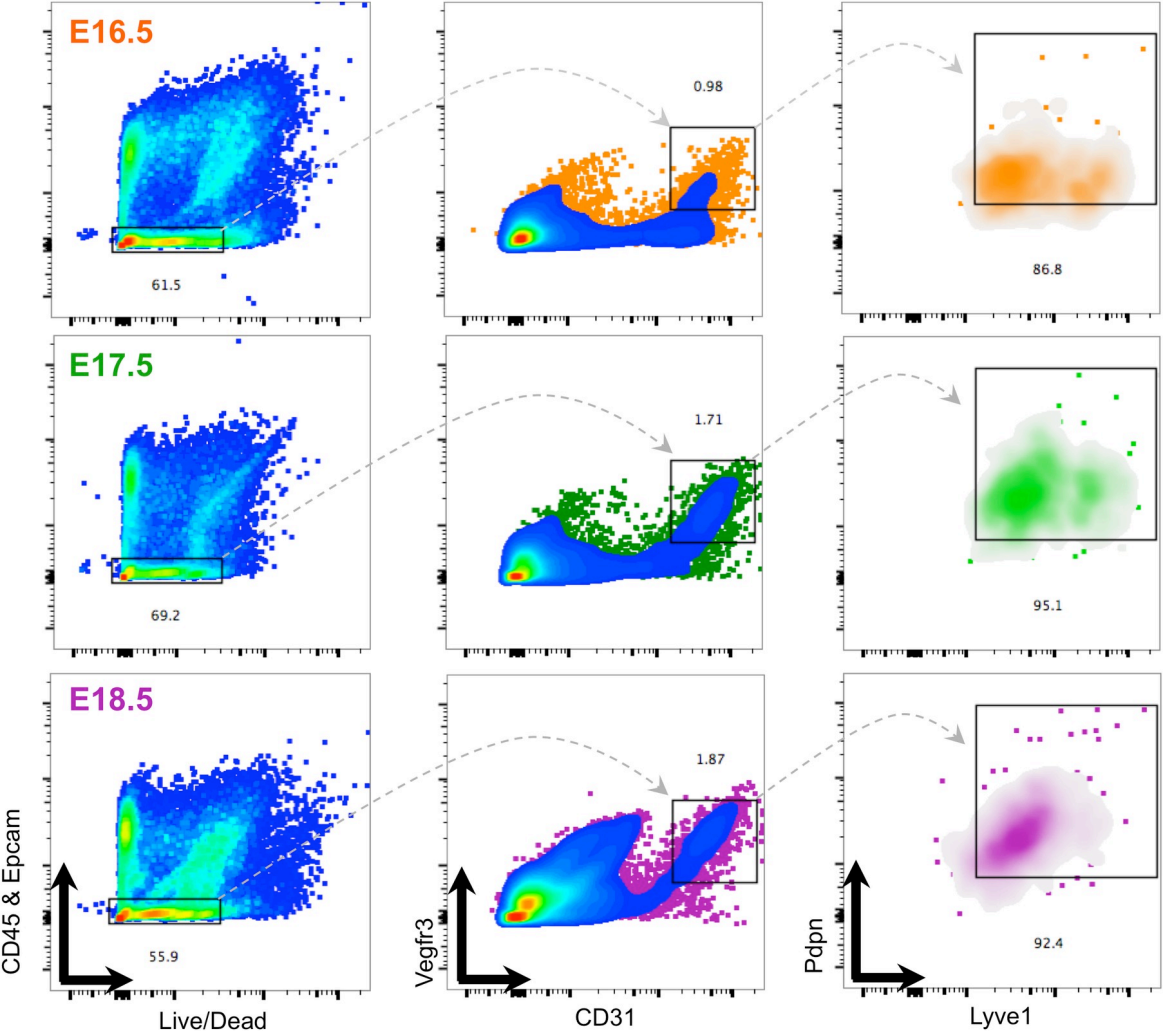
Step-wise gating strategy for isolation of PLECs (CD45-/Epcam-/CD31+/Vegfr3+/Lyve1+/Pdpn+) from late-gestation fetal lungs. Representative gate for live, CD45- and Epcam- lung cells is boxed (*left panels*). The gate containing the CD45-/Epcam-/CD31+/Vegfr3+ population (boxed, middle panels). (*Right panels*) The CD45-/Epcam-/CD31+/Vegfr3+ cellular fraction expressed both Pdpn+ and Lyve1+ characteristic of the lymphatic endothelial phenotype across late-gestation (boxed, *right panels*).

To examine this further, we collected mRNA and performed qRT-PCR to measure the expression of lymphatic endothelial markers in sorted lung fractions. Compared to other lung cell fractions, the expression of *Prox1* and *Flt4/Vegfr3* was significantly higher in sorted CD31+/Vegfr3+/Pdpn+/Lyve1+ PLECs at E18.5 (Fig 2B). Importantly, *Emcn*, a specific marker of blood endothelial cells was expressed at significantly higher levels in the CD31+/Vegfr3- fraction than in sorted E18.5 PLECs (Fig 2B). Furthermore, the expression of *Prox1*, *Flt4/Vegfr3*, and *Lyve1* was increased 108-fold, 48-fold, and 8-fold, respectively, in isolated PLECs compared to whole E18.5 lung (Fig 3). Fig 4 shows the sequential gating strategy used to isolate CD31+/Vegfr3+/Pdpn+/Lyve1+ PLECs from E16.5, E17.5 and E18.5 fetal lungs. Taken together, these data indicate that RNA collected from sorted CD31+/Vegfr3+/Pdpn+/Lyve1+ cells can be used to characterize the transcriptional programs underlying prenatal PLEC maturation prior to the birth transition to air breathing.

### Temporal-dependent patterns of gene expression in late-gestation PLECs

**Fig 5:**
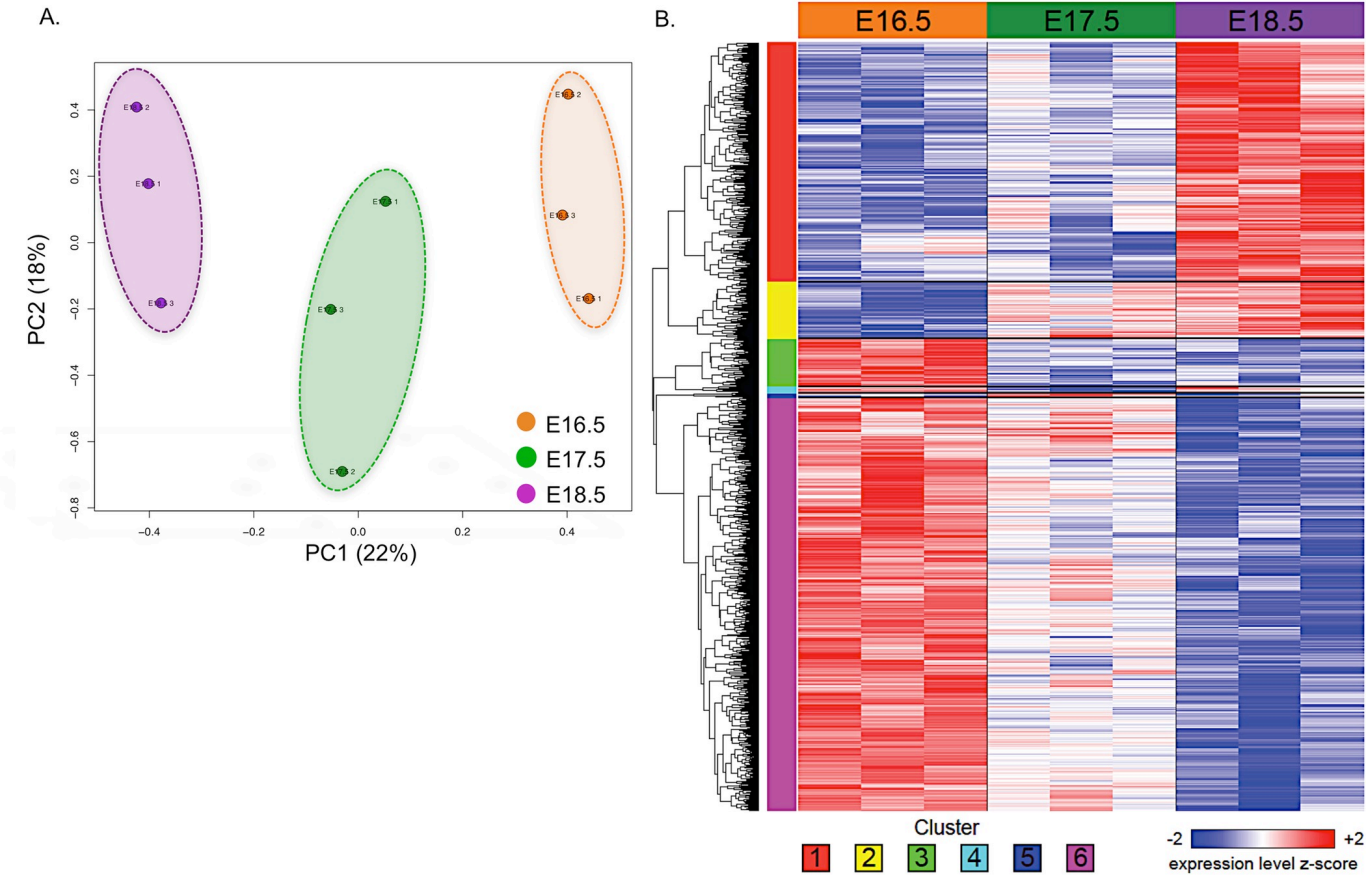
Temporal-dependent gene expression in fetal PLECs. (A) Principal component analysis of E16.5, E17.5, and E18.5 sorted PLECs (n = 3 per time point). Each axis indicates the fraction of experimental variance explained by each principal component (PC). (B) Heatmap of all 1,281 genes with significant differential expression with respect to time (ANOVA FDR *q* < 0.05). The number of each cluster is indicated at the bottom of the heatmap. Expression values for each gene were *z*-normalized to a mean of zero and standard deviation of one across all samples in each row; blue, white, and red indicate final *z*-scores of ≤ -2, 0, and ≥ 2, respectively.

We then used whole-genome microarrays to profile gene expression in RNA samples isolated from sorted CD31+/Vegfr3+/Lyve1+/Pdpn+ PLECs at E16.5, E17.5, and E18.5 days of gestation (n = 3 per time point). Principal Component Analysis (PCA) performed across all genes showed that the samples separated strongly with respect to time along the first principal component (PC1), which explains 22% of total variance (Fig 5A), indicating that many genes are up- or down-regulated in a progressive manner from E16.5 to E17.5 to E18.5. A one-way analysis of variance (ANOVA) was then performed to identify the predominant patterns of coordinate differential expression with respect to time, and *t* tests were performed for each gene between the E16.5 and E18.5 time points (S1 File and Tables 1 and 2). After excluding genes that were not expressed above the median value of at least one array, 1,281 genes, whose expression was significantly associated with time (ANOVA, FDR *q* < 0.05), were partitioned into six clusters (Fig 5B and S2 File).

**Table 1:** Classification of genes upregulated in E18.5 vs. E16.5 PLECs (FC≥2, p<0.05, FDR q<0.25).

	FC	pVAL	FDR		FC	pVAL	FDR
**Interferon alpha & beta signaling**				**Extracellular matrix**			
*Ifit1*	7.4	1.0E-05	3.1E-03	*Chi3l1*	9.6	1.0E-05	3.1E-03
*Usp18*	7.2	3.4E-06	2.1E-03	*Cp*	8.5	2.3E-04	1.0E-02
*Mx2*	6.9	5.2E-05	5.3E-03	*Spon1*	6.9	3.8E-05	4.5E-03
*Sp100*	6.8	6.5E-06	2.9E-03	*Serpina3n*	5.5	9.2E-05	6.6E-03
*Ifi44*	6.7	2.0E-06	2.0E-03	*Scube2*	4.9	1.7E-03	2.9E-02
*Irf7*	6.1	5.1E-05	5.3E-03	*Pcdhac2*	4.5	8.1E-04	1.9E-02
*Iigp1*	6.1	1.2E-06	1.9E-03	*Has1*	4.5	1.9E-05	3.5E-03
*Oasl2*	5.7	3.5E-06	2.1E-03	*Pcolce2*	4.2	2.7E-04	1.1E-02
*Isg15*	4.1	2.8E-04	1.1E-02	*Adamtsl2*	3.7	4.7E-04	1.4E-02
*Oas2*	3.8	1.9E-04	9.4E-03	*Fn1*	3.1	8.0E-04	1.9E-02
*Ifih1*	3.8	9.5E-06	3.1E-03	*Eln*	2.5	5.2E-03	5.1E-02
*Ifi47*	3.6	7.6E-05	6.2E-03	*Egflam*	2.3	5.2E-03	5.1E-02
*Ifi203*	3.5	9.0E-05	6.6E-03				
*Isg20*	3.2	2.0E-03	3.1E-02	**Cell adhesion & cell junction**			
*Igtp*	3.0	6.8E-05	6.0E-03	*Dpt*	9.6	4.0E-05	4.7E-03
*Oasl1*	2.5	2.7E-04	1.1E-02	*Lgals3bp*	6.0	8.9E-06	3.1E-03
				*Dtna*	4.2	7.2E-05	6.1E-03
**Angiogenesis and vascular development**				*Gsn*	4.1	8.9E-05	6.5E-03
*Ntrk2*	4.8	7.9E-05	6.3E-03	*Selp*	3.3	1.6E-02	9.6E-02
*Xdh*	4.2	6.4E-05	5.8E-03	*Spock2*	2.9	7.2E-06	2.9E-03
*Sema3c*	3.3	5.0E-04	1.5E-02	*Itgb3*	2.9	7.9E-03	6.5E-02
*Fgf1*	2.9	2.8E-05	3.8E-03	*Itga8*	2.7	2.4E-03	3.4E-02
*Pdpn*	2.8	5.0E-04	1.5E-02	*Itga2b*	2.7	1.5E-02	9.2E-02
*Vegfa*	2.7	5.3E-05	5.3E-03	*Itgb5*	2.7	2.5E-04	1.1E-02
*Srpx2*	2.7	1.2E-03	2.3E-02	*Itgb2*	2.7	7.7E-03	6.4E-02
*Fgfr4*	2.6	5.3E-03	5.2E-02	*Tgm1*	2.6	6.3E-05	5.8E-03
*Sphk1*	2.6	2.1E-04	9.8E-03	*Fermt3*	2.4	3.3E-02	1.4E-01
*Enpep*	2.5	9.1E-03	7.0E-02	*Npnt*	2.3	1.1E-02	7.8E-02
*Fgfr3*	2.5	1.9E-03	3.0E-02				
*Ang*	2.4	2.8E-02	1.3E-01	**Receptors and kinases**			
*Ccbe1*	2.4	7.2E-05	6.1E-03	*Ntrk2*	4.8	7.9E-05	6.3E-03
*Figf*	2.1	3.2E-02	1.4E-01	*Ccbp2*	3.8	1.3E-03	2.4E-02
				*Akap5*	3.0	4.0E-05	4.7E-03
**Complement & coagulation cascade**				*Itpkc*	2.8	8.7E-05	6.5E-03
*C7*	17.6	3.7E-05	4.5E-03	*Fgfr4*	2.6	5.3E-03	5.2E-02
*Hc*	8.1	7.1E-06	2.9E-03	*Sphk1*	2.6	2.1E-04	9.8E-03
*Serpine1*	5.0	7.9E-05	6.3E-03	*Fgfr3*	2.5	1.9E-03	3.0E-02
*Bdkrb2*	4.3	7.8E-03	6.4E-02	*Nrbp2*	2.4	3.3E-04	1.2E-02
*Serping1*	3.7	5.7E-04	1.6E-02	*Vipr2*	2.3	1.2E-04	7.1E-03
*Clec3b*	3.5	7.1E-05	6.1E-03	*S1pr3*	2.2	3.3E-02	1.4E-01
*F2rl2*	3.5	5.8E-02	1.9E-01				
*C3*	3.2	1.2E-02	8.2E-02	**Transcriptional regulators**			
*Clec1b*	2.9	6.9E-02	2.1E-01	*Sp100*	6.8	6.5E-06	2.9E-03
*Cfh*	2.6	5.6E-03	5.4E-02	*Irf7*	6.1	5.1E-05	5.3E-03
*Tfpi2*	2.1	4.4E-02	1.6E-01	*Fhl5*	6.0	4.1E-02	1.6E-01
				*Trim30a*	5.2	3.4E-05	4.3E-03
**Lipid binding, synthesis, metabolism**				*Batf2*	4.1	1.8E-03	2.9E-02
*Rsad2*	8.1	4.7E-04	1.5E-02	*Nfe2*	3.2	9.2E-03	7.0E-02
*Scd1*	6.6	1.2E-07	8.4E-04	*Ddx60*	3.2	4.7E-03	4.9E-02
*Hsd11b1*	4.3	1.1E-03	2.2E-02	*Tcf21*	3.2	2.2E-02	1.1E-01
*Olr1*	4.2	9.7E-07	1.9E-03	*Aff3*	3.1	2.4E-05	3.6E-03
*Ch25h*	3.6	5.8E-03	5.5E-02	*Daxx*	2.4	4.9E-04	1.5E-02
*Fabp4*	3.5	4.0E-03	4.4E-02				
*Ces1d*	2.7	1.2E-03	2.3E-02				
*Aldh2*	2.6	4.2E-04	1.4E-02				
*Liph*	2.6	6.3E-04	1.6E-02				
*Plscr1*	2.2	1.9E-04	9.4E-03				
*Lipe*	2.1	1.3E-03	2.4E-02				
*Lpl*	2.1	6.2E-04	1.6E-02				
*Plscr4*	2.0	2.9E-05	4.0E-03				
*Echdc2*	2.0	7.4E-03	6.3E-02				

**Table 2:** Classification of genes downregulated in E18.5 vs. E16.5 PLECs (FC≤-2, p<0.05, FDR q<0.25).

	FC	Pval	FDR		FC	Pval	FDR
**Cell Cycle**				**mRNA Processing**			
*Mcm5*	-4.1	8.6E-05	6.5E-03	*Tmem48*	-3.5	5.2E-07	1.8E-03
*Tipin*	-4.0	1.6E-05	3.5E-03	*Raet1d*	-3.4	5.7E-03	5.4E-02
*Fbxo5*	-3.8	1.6E-04	8.7E-03	*Rrm2*	-3.3	2.5E-05	3.6E-03
*Bub1*	-3.7	5.1E-05	5.3E-03	*Pmf1*	-3.2	1.8E-05	3.5E-03
*Kif11*	-3.4	6.8E-05	6.0E-03	*Exosc6*	-2.7	5.6E-03	5.3E-02
*Cenph*	-3.3	1.1E-06	1.9E-03	*Rnaseh2b*	-2.4	1.7E-05	3.5E-03
*Chek1*	-2.9	3.5E-05	4.4E-03				
*Mki67*	-2.4	3.2E-03	4.0E-02	**Ubiquitin proteasome pathway**			
*Iqgap3*	-2.0	4.6E-03	4.8E-02	*Dtl*	-3.3	3.4E-06	2.1E-03
				*Skp2*	-3.1	5.0E-05	5.3E-03
**DNA Replication**				*Usp1*	-2.6	2.3E-04	1.0E-02
*Pole*	-4.4	3.2E-05	4.2E-03	*Uhrf1*	-2.5	4.9E-04	1.5E-02
*Rfc4*	-3.8	4.8E-06	2.4E-03	*Uchl5*	-2.3	7.6E-04	1.8E-02
*Rad51*	-3.4	1.2E-04	7.1E-03	*Ube2c*	-2.1	1.9E-04	9.4E-03
*Cenph*	-3.3	1.1E-06	1.9E-03				
*Dtl*	-3.3	3.4E-06	2.1E-03	**Transcriptional regulators**			
*Chaf1b*	-3.2	1.1E-04	6.9E-03	*E2f8*	-3.6	1.6E-04	8.6E-03
*Lig1*	-3.2	3.7E-06	2.1E-03	*Tacc3*	-2.9	2.3E-05	3.6E-03
				*E2f1*	-2.8	7.8E-04	1.8E-02
**Oxidoreductase Activity**				*Foxp2*	-2.7	3.7E-02	1.5E-01
*Cyp1a1*	-3.3	6.3E-03	5.7E-02	*Foxm1*	-2.6	1.0E-04	6.9E-03
*Dhfr*	-3.2	2.2E-05	3.5E-03	*Cdk4*	-2.4	5.7E-04	1.6E-02
*Mthfd2*	-2.3	7.4E-04	1.8E-02	*Rbl1*	-2.2	7.8E-05	6.3E-03
*Srd5a1*	-2.2	1.9E-04	9.4E-03	*Trp53*	-2.2	1.0E-05	3.1E-03
*Dhcr7*	-2.0	2.5E-04	1.1E-02				
				**Receptors and receptor interactors**			
**Chromosome maintenance**				*Hmmr/Rhamm*	-3.0	2.5E-04	1.1E-02
*Hells*	-5.5	3.5E-06	2.1E-03	*Lpar4*	-2.7	1.2E-04	7.1E-03
*Prim1*	-4.9	3.8E-06	2.1E-03	*Trip13*	-2.3	2.4E-03	3.4E-02
*Mcm7*	-3.7	3.7E-06	2.1E-03	*Lpar6*	-2.2	1.7E-03	2.8E-02
*Smc2*	-3.6	1.6E-04	8.6E-03	*Traf4*	-2.0	4.1E-03	4.5E-02
*Mcm2*	-3.2	2.6E-05	3.7E-03	*Mc5r*	-2.0	3.2E-02	1.4E-01
*Dkc1*	-2.4	2.8E-04	1.1E-02				
**Cell adhesion & cell junction**							
*Stab2*	-2.9	2.2E-04	1.0E-02				
*Cldn6*	-2.3	1.3E-02	8.7E-02				
*Vcan*	-2.3	2.4E-02	1.2E-01				
*Cdh2*	-2.1	3.2E-04	1.2E-02				

Cluster 1 is comprised of 398 genes that are slightly upregulated from E16.5 to E17.5 and then strongly upregulated at E18.5. DAVID analysis (results for all clusters are in S3 and S4 Files) indicated that this cluster is strongly and significantly enriched in genes associated with the GO terms "defense response to virus" (GO:0051607, FDR *q* = 7.6x10^-6^), which includes *Cxcl10*, *Dhx58*, *Gpam*, *Ifit1*, *Il33*, *Il6*, *Isg15*, *Isg20*, *Mx2*, *Oas2*, *Oasl1*, *Oasl2*, *Plscr1*, *Rnasel*, *Rsad2*, *Samhd1*, *Stat2*, and *Zc3hav1*; "cellular response to interferon-beta" (GO:0035458, FDR *q* = 1.7x10^-4^), which includes *Gbp2*, *Gbp3*, *Gm4951*, *Ifi203*, *Ifi47*, *Ifit1*, *Igtp*, *Iigp1*, and *Irgm1*; "response to cytokine" (GO:0034097, FDR *q* = 3.6x10^-3^), which includes *Cd274*, *Cxcl16*, *Mapkapk3*, *Ptgs2*, *Scgb1a1*, *Serpina3f*, *Serpina3g*, *Serpina3n*, *Skil*, and *Timp2;* "cell adhesion" (GO:0007155, FDR *q* = 6.7x10^-6^); "extracellular matrix organization" (GO:0030198, FDR *q* = 1.1x10^-5^); and "positive regulation of angiogenesis" (GO:0045766, FDR q = 1.2x10^-4^), including *Anxa3*, *C3*, *Ccbe1*, *Chi3l1*, *Ctsh*, *Ecm1*, *Epha1*, *Hc*, *Hspb1*, *Sema5a*, *Serpine1*, *Sphk1*, *Thbs1*, and *Vegfa*. This cluster also showed nominally significant enrichment for the two overlapping GO terms "fatty acid metabolic process" (GO:0006631, *p* = 9.8x10^-3^) and "lipid metabolic process" (GO:0006629, *p* = 0.029), which include *Abhd5*, *Apoe*, *C3*, *Ces1d*, *Echdc2*, *Fabp4*, *Fads3*, *Gpam*, *Hsd11b1*, *Liph*, *Lpin2*, *Lpl*, *Pmvk*, *Ptgs2*, *Scd1*, *Sftpb*, and *Vldlr*.

Cluster 2 contains 95 genes that are upregulated in a gradual manner from E16.5 to E17.5 and then to E18.5. These genes are significantly associated with the GO terms "positive regulation of antigen processing and presentation of endogenous peptide antigen via MHC class I" (GO:0019885, *p* = 1.8x10^-4^, FDR q = 0.042), including *Tap1*, *Tap2* and *Tapbp*, and "innate immune response" (GO:0045087, *p* = 1.7x10^-3^, FDR *q* = 0.21), including *Adar*, *Ifih1*, *Irf7*, *Nod2*, *Pml*, *Tbkbp1*, *Trim21*, and *Trim25*.

Cluster 3 encompasses 80 genes with an expression pattern that is abruptly down-regulated between E16.5 and E17.5 and remains low at E18.5, including genes involved in ubiquitin-dependent protein catabolism (GO:0006511, *p* = 0.020): *Psma1*, *Psma4*, *Ube2c*, and *Usp11*. Surprisingly, cluster 4, which contains only 11 genes with a trough-like pattern of down-regulation at E17.5 followed by reversion to E16.5 levels at E18.5, was significantly associated with the GO term "steroid metabolic process" (GO:0008202, FDR *q* = 0.066) as it contains the genes *Hmgcs1*, *Sc4mol*, and *Soat1*. However, cluster 5, which contains only eight genes that peak at E17.5, was too small to achieve statistical significance for any GO terms.

Cluster 6 contains 689 genes that are progressively down-regulated from E16.5 to E17.5 and then to E18.5. This cluster is significantly enriched in genes that are involved in mitotic processes and are associated with GO terms such as "cell cycle" (GO:0007049, *p* = 2.3 x 10^−68^), e.g., *Brca1*, *Ccna2*, *Ccnb2*, *Ccne1/2*, *Ccnf*, *Cdc6/7*, *Cdc25a/c*, *Cdk1/2/4*, *Chek1/2*, *Mcm4/5*, and *Mki67*; "DNA replication" (GO:0006260, *p* = 6.9 x 10^−36^), e.g., *Cdt1*, *Chaf1a/b*, *Gins1/2/4*, *Lig1*, *Pole*, *Polh*, *Prim1/2*, *Ticrr*; and "mRNA processing" (GO:0006397, *p* = 5.1x10^-9^), e.g., *Cpsf2/3*, *Dhx20/Dhx39*, *Gemin5*, *Khsrp*, *Lsm2*, *Rbmx*, *Rnps1*, *Ttf2*, *Usp39*, *Wdr33* and *Zcchc8*.

### Gene set enrichment analysis

**Fig 6:**
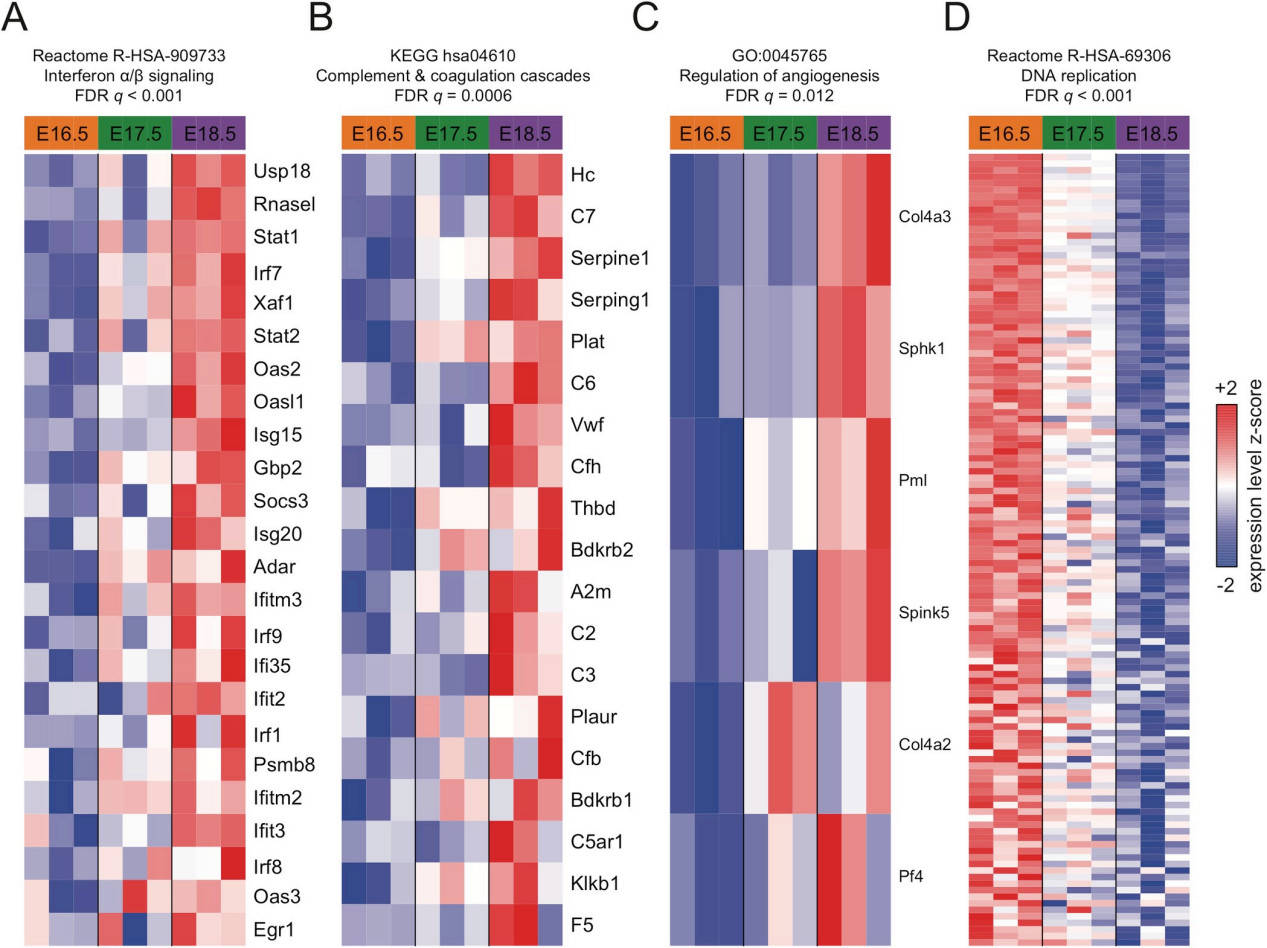
Leading edge heatmaps of selected GSEA results. The expression of the leading edge genes of each GSEA result is shown for (A) Reactome "interferon α/β signaling", (B) KEGG "complement and coagulation cascades", (C) Gene Ontology "regulation of angiogenesis", and (D) Reactome "DNA replication". Genes (rows) are arranged in descending order by the magnitude of the E18.5 vs E16.5 *t* statistic. Expression values for each gene were *z*-normalized to a mean of zero and standard deviation of one across all samples in each row; blue, white, and red indicate final *z*-scores of ≤ -2, 0, and ≥ 2, respectively.

Although the clustering analysis described above identified numerous pathways that are significantly overrepresented in gene clusters with temporal-dependent expression, some of these results are derived from a relatively small number of genes. To increase statistical power to identify biological pathways that are coordinately up- or down-regulated with respect to time, we next performed Gene Set Enrichment Analysis (GSEA), ranking the human homologs of all genes interrogated by the microarray according to the *t* statistic computed between the early and late time points. The full results of the GSEA analysis are included in S5 File. Many gene sets were significantly coordinately upregulated from E16.5 to E18.5, including the Reactome "interferon alpha/beta signaling" pathway (R-HSA-909733, FDR *q* < 0.001), which includes *Socs3*, *Irf7*, *Stat1*, *Stat2*, and *Usp18* (Fig 6A); the KEGG pathway "complement and coagulation cascades" (hsa04610, FDR *q* = 0.0006), which contains the *Serping1* and *Serpine1*, *two* genes with fibrinolytic and plasminogen inhibitor activity; and the complement genes *C3*, *C7*, *Cfh* and *Hc (*Fig 6B); and the GO term "regulation of angiogenesis" (GO:0045765, FDR *q* = 0.012) were enriched in genes upregulated at E18.5 (Fig 6C and Table 1).

Conversely, many gene sets related to mitotic cell cycle were significantly coordinately down-regulated from E16.5 to E18.5, including the Reactome "DNA replication" pathway (R-HSA-69306, FDR *q* < 0.001), which includes *Mcm5*, a gene involved in licensing DNA replication (Fig 6D and Table 2).

**Fig 7:**
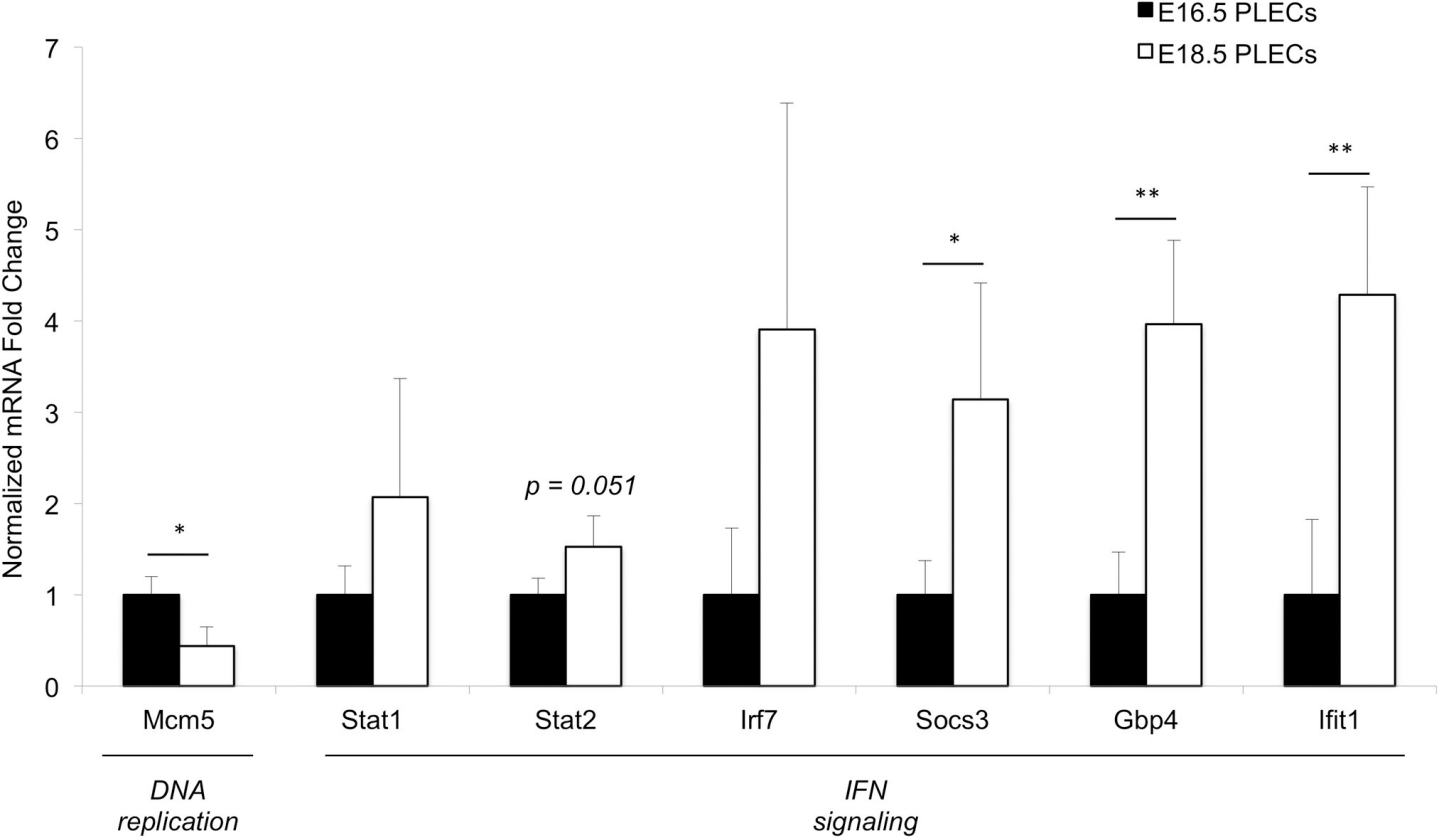
Down-regulation of cell-cycle and upregulation of IFN pathway genes in fetal PLECs. qRT-PCR analysis of gene expression in independently sorted PLEC samples, (E16.5, *n = 3*; E18.5, *n = 2* or *n = 3* for *Ifit1* and *Irf7*). Statistical analysis was performed using an unpaired 1-tailed Student *t* test (**p<0.*01*, *p<0.*05*).

**Fig 8:**
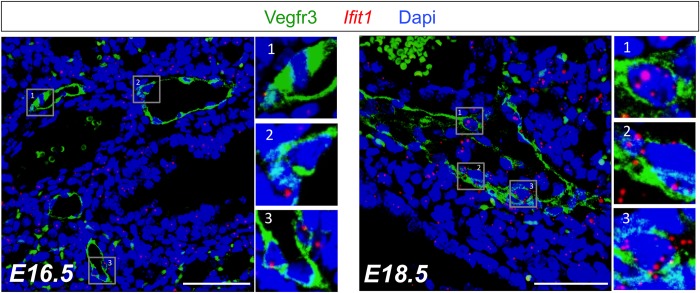
Increased expression of *Ifit1* mRNA in E18.5 PLECs *in vivo*. Single molecule fluorescent *in situ* hybridization for *Ifit1* mRNA (red punctae) and Vegfr3 (green) immunostaining in E16.5 and E18.5 lungs. Boxed, numbered insets have been cropped, magnified and shown at the right of each image. Note the increased red punctae representing *Ifit1* mRNA in Vegfr3+ PLECs at E18.5. (*scale bar*, *50 um*).

We selected several canonical interferon-stimulated genes (ISGs) and other genes involved in the IFN-I pathway and confirmed their upregulation in independently sorted E16.5 and E18.5 PLEC samples by qRT-PCR. We also confirmed down-regulation of *Mcm5* in E18.5 PLECs (Fig 7). Next we used single-molecule fluorescent *in situ* hybridization probes against *Ifit1* (interferon-induced protein with tetratricopeptide repeats 1), a well-established ISG significantly upregulated from E16.5 to E18.5 (7.4-fold, FDR *q* = 3.1x10^-3^) to further confirm that the IFN-I pathway was activated in E18.5 PLECs *in vivo* (Fig 8). This analysis demonstrated increased expression of *Ifit1* in Vegfr3+ LECs at E18.5 versus E16.5. Combined with the microarray and qRT-PCR analysis, these data provide further evidence that PLECs are a target of IFN-I signaling in late gestation and that ISGs are transcriptionally active in fetal LECs *in vivo*.

### Fetal PLEC specific maturation profile

**Fig 9:**
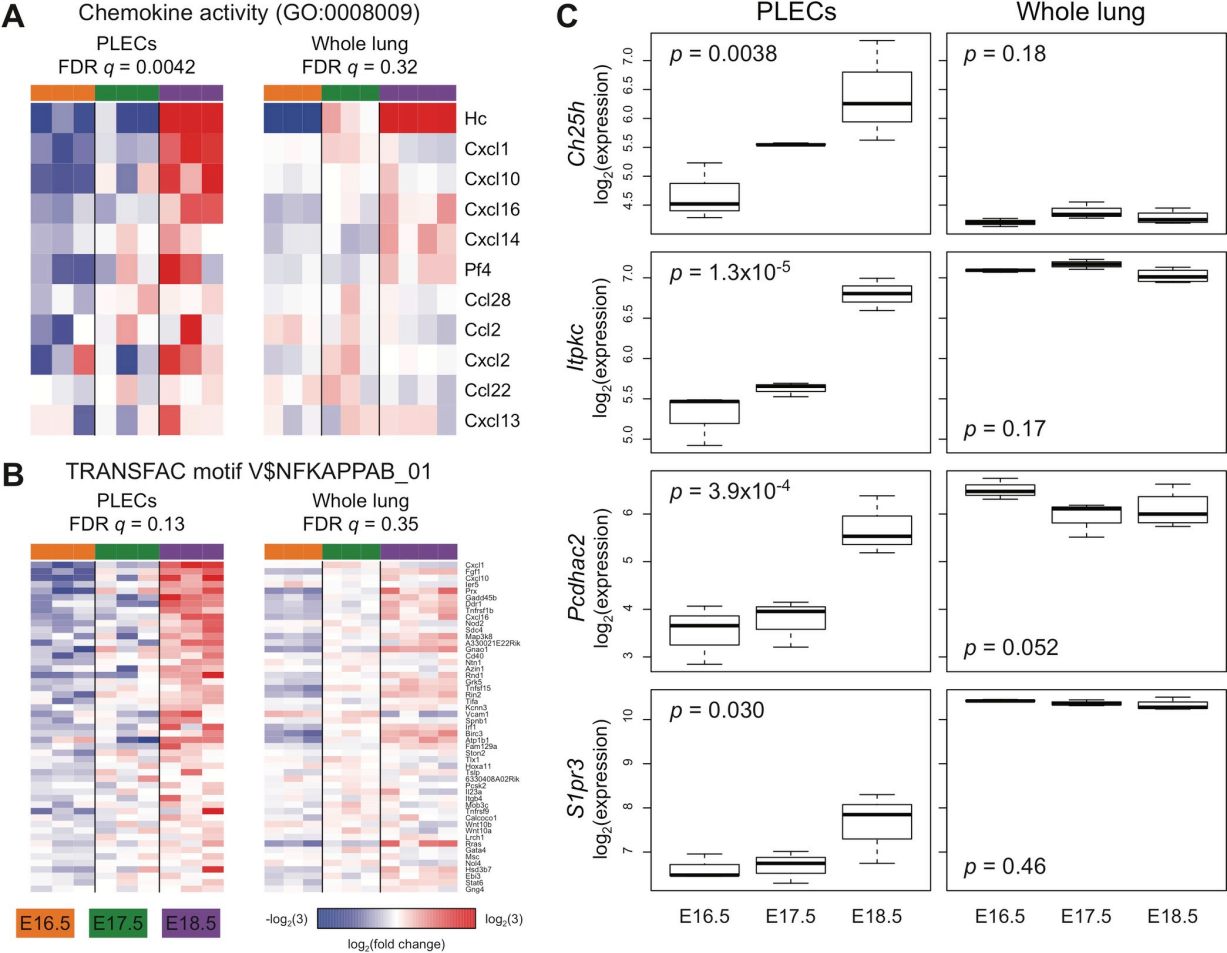
PLEC-specific transcriptional events during fetal lung maturation. Gene Set Enrichment Analysis (GSEA) demonstrated that genes annotated with the GO term "chemokine activity" (GO:0008009) (A) or NF-κB target genes (TRANSFAC motif V$NFKAPPAB_01) (B) were significantly (FDR *q* < 0.25) coordinately upregulated during fetal maturation in PLECs but not in whole lung (GEO Series GSE35485). Expression of the genes in the leading edge of each PLEC GSEA result is shown in both datasets, with red and blue indicating values up- or down-regulated, respectively, at least 3-fold from the mean (white) across all samples within each dataset (A-B). (C) The expression of select genes changes significantly (one-way ANOVA *p* < 0.05) with respect to time in sorted PLECs (upregulated from E16.5 to E18.5) but not in the whole lung.

To identify genes and pathways that are unique to PLECs during prenatal maturation, we repeated the statistical analysis and GSEA using a publicly available microarray dataset (GSE35485) profiling whole fetal lung in C57BL/6 animals at the same time points, and compared these results with those obtained from the PLECs. The full set of statistical results is provided in S6 File, and a tabulation of the E18.5 vs E16.5 GSEA results in both the PLECs and GSE35485 is provided in S7 File. These analyses demonstrated that sets of genes annotated with the GO term "chemokine activity" (GO:0008009) or containing an NF-κB binding site (TRANSFAC motif V$NFKAPPAB_01) in their promoter were significantly upregulated between E16.5 and E18.5 (FDR *q* < 0.25) in PLECs but not in whole lung. The “chemokine activity” gene set included *Cxcl1*, *Cxcl10*, *Cxcl16*, *Cxcl2*, and *Pf4/Cxcl4*, and “NF-κB target genes” included *Prx*, *Nod2*, *Ddr1*, *Tnfrsf1b*, *Sdc4*, *Ntn1* and *Azin1* (Fig 9A and 9B). In addition, the expression of several genes listed in Table 1 changed significantly with respect to time in PLECs (one-way ANOVA *p* < 0.05) but not in whole lung, including *Ch25h*, *Itpkc*, *Pcdhac2* and *S1pr3*, each of which were upregulated from E16.5 to E18.5 in a PLEC-specific manner (Fig 9C). Overall, our results highlight both broad and PLEC-specific maturation profiles during late gestation lung development.

## Discussion

The switch from placental to pulmonary gas exchange at birth requires fluid removal and the capacity to face the immunologic challenges of air breathing. An intact and mature pulmonary lymphatic system is essential for each of these fundamental processes. In this study we developed and implemented a cell sorting strategy that enabled the first transcriptional profiling of PLECs during late gestation.

GSEA was used to identify pathways that are coordinately regulated from E16.5 to E18.5 fetal time points. This analysis demonstrated that genes involved in IFN signaling are a salient feature of maturing pulmonary lymphatics, which was confirmed by qRT-PCR and *in situ* hybridization. Transcriptional activation of IFN target genes at E18.5 indicates that PLECs act as a receiver of IFN signaling during late gestation. Although the specific effects of IFN on lymphatic formation and function in the lung have not been definitively studied, the prevailing concept is that IFNs inhibit both angiogenesis and lymphangiogenesis in postnatal life [27–30]. Importantly, others have linked IFNα signaling to maturation of fetal hematopoietic stem cells [31]. Taken together, these findings are consistent with a role for IFN signaling in maturation of the pulmonary lymphatic endothelium prior to birth.

Genes involved in angiogenesis were upregulated in PLECs from E16.5 to E18.5. One key molecule is sphingosine kinase 1 (Sphk1), which is required for the production of sphingosine-1-phosphate (S1P), a lipid important for angiogenesis, vascular maintenance, LEC maturation, as well as lymphocyte mobilization [32, 33]. Another important gene for lymphangiogenesis, *Ccbe1* was upregulated in E18.5 PLECs, and this gene is a Vegf-C potentiator, and thus mutations in Ccbe1 result in lymphatic malformation, lymphedema and other complex phenotypes associated with Hennekam syndrome [34–37].

Genes involved in the complement and coagulation cascade were upregulated in E18.5 PLECs. For proper interstitial fluid clearance and leukocyte trafficking, lymph must be a hypo-coagulable biological fluid [38, 39]. Consistent with this, we observed increased expression of anti-coagulative genes (*Serpine1* and *Serping1*), however genes known to promote coagulation (*F2rl2*, *Tfpi2*, and *Clec1b/3b)* were also induced in E18.5 PLECs. Coagulation and clot formation play an important role in lymphatic biology throughout life [40–42], but a role for complement factors in lymphatic maturation and function has not been described.

The lymphatic endothelium possesses several highly specialized features that make them unique and optimally adapted for clearance of interstitial fluids, macromolecules, and for leukocyte transmigration. For instance, capillary LECs contain incomplete basement membranes devoid of pericyte investment, and exhibit discontinuous intercellular junctions termed “button-junctions;” essentially an entry point for fluids, macromolecules and leukocytes [18, 43]. Notably, our analysis identified a cluster of genes that are significantly upregulated at E18.5, which encode for proteins critical to “cell adhesion”, “cell-cell junctions” and interaction with the “extracellular matrix”. This cluster also contains the surface receptor *Fgfr3*, a direct target of Prox1 [44], which is critical for lymphatic development, growth and regulation of LEC metabolism [45]. Other genes in this cluster include *Ccbp2*/D6, which is expressed by LECs to influence adaptive immunity [46]. Transcription factors such as *Sp100*, a tumor suppressor known to regulate endothelial migration and angiogenesis [47]; *Trim30a*, a TLR-responsive inhibitor of NF-kB signaling [48] and *Irf7*, the master regulator of IFN-I mediated immune protection [49] were all upregulated in E18.5 PLECs. Furthermore, there was upregulation of *Pdpn*, which is required for proper lymphatic-venous separation during development and for dendritic cell migration to lymph nodes in postnatal life [50]. *Sema3c*, a member of the Semaphorin 3 family involved in lymphatic maturation and valve formation was upregulated at E18.5 [51]. GSEA and cluster analyses showed down-regulation of genes involved in cell cycle regulation and DNA replication pathways, suggestive of cellular maturation or terminal differentiation of the pulmonary lymphatic endothelium prenatally [52]. And lastly, recent evidence indicates a major role for fatty acid β-oxidation and lipid metabolism in lymphatic development [53]. Our data are in line with these findings since the microarray analysis detected increased expression of genes involved in lipid biosynthesis and metabolism in E18.5 PLECs suggesting a link between lipid signaling and prenatal pulmonary lymphatic maturation.

Gene sets such as “*chemokine activity*” and “*NF-kB targets*” were enriched specifically in E18.5 PLECs. It is noteworthy that genes with NF-kB binding sites in their promoters were enriched in PLECs during late fetal gestation because constitutive NF-kB activity has previously been shown in lymphatic beds of multiple organs in adult mice [54]. Both *Cxcl1* and *Cxcl10*, ISGs which regulate chemotaxis and leukocyte trafficking [55] were represented in both PLEC specific gene sets and in type-I IFN signaling. Other upregulated genes unique to PLECs were *Ch25h* and *S1pr3*. Ch25h is involved in catabolism of cholesterol and leads to generation of oxysterol, a ligand for EBI2, a receptor that regulates chemotaxis and positioning of lymphoid lineages in lymph nodes [56]. *S1pr3*, a gene upregulated specifically in PLECs promotes lymphoid mobilization from lymphoid tissues [57]. Overall, our analyses identify broad mechanisms and PLEC specific signatures of maturation during late gestational lung development.

## Conclusions

Our microarray analysis indicated the repression and activation of numerous fundamental processes during late-gestation lung development and suggests the presence of a distinct prenatal maturation program for fetal PLECs. Further studies are needed to determine the precise contribution of IFN signaling or alternative pathways to lymphatic function and postnatal pulmonary biology. The importance of lung lymphatics is demonstrated by their involvement in immunity, fluid clearance and air breathing at birth. This work reveals transcriptional events associated with PLEC maturation prior to birth and may provide some insight into the dysfunction and pathology of the premature lung.

## Supporting information

S1 File Statistical analysis of PLEC gene expression microarray experiment. Each row corresponds to a single mouse Entrez Gene. Columns A-E contain the MBNI probeset identifier, links to the Entrez Gene record(s) for the mouse gene and any of its human homolog(s), and the gene symbol and description as obtained from version 17.0.0 of the MBNI mogene20stmmentrezg.db R package. Columns F-G contain the nominal *p* value and FDR *q* value from the moderated one-way ANOVA, and columns H-K contain the signed fold change (computed in linear space), moderated *t* statistic, nominal *p* value, and FDR *q* value for the comparison between the E18.5 and E16.5 time points. All FDR *q* values were computed after excluding any genes that were not expressed above the median value of at least one of all nine samples. Columns L-T contain the log2 (expression) of each gene in all samples, shaded so that blue and red indicate expression values that are at least two standard deviations below and above, respectively, the mean (white) of each row. The rows are sorted in ascending order by one-way ANOVA *p* value.
(XLSX)

S2 File Hierarchical clustering analysis. Each row corresponds to a single mouse Entrez Gene. Columns A-E contain the MBNI probeset identifier, links to the Entrez Gene record(s) for the mouse gene and any of its human homolog(s), and the gene symbol and description as obtained from version 17.0.0 of the MBNI mogene20stmmentrezg.db R package. Column F contains the cluster number. Columns G-O contain the log2 (expression) of each gene in all samples, shaded so that blue and red indicate expression values that are at least two standard deviations below and above, respectively, the mean (white) of each row. The rows are arranged in the same order as the hierarchical clustering heatmap in Fig 5B.
(XLSX)

S3 File Results of DAVID functional enrichment analysis. Each row corresponds to the result provided by DAVID for a single Gene Ontology (GO) term and a single cluster from the hierarchical clustering analysis. Column A indicates the cluster, and columns B-C indicate the GO term identifier and name. Columns D and E indicate the nominal *p* value and the Benjamini-Hochberg FDR *q* value for each DAVID result within each cluster. Column F indicates the gene symbols corresponding to the Entrez Gene identifiers that were present in both the cluster and the GO term.
(XLSX)

S4 File Summary of genes included in selected DAVID results. Each tab of the file summarizes the results for selected GO terms for a specific gene cluster. Column A contains the gene symbol, column B contains the signed fold change (computed in linear space) for the comparison between the E18.5 and E16.5 time points, and columns C and D contain the nominal *p* value and FDR *q* value from the moderated one-way ANOVA, where the FDR *q* values were computed after excluding any genes that were not expressed above the median value of at least one of all nine samples. Each set of genes is sorted in descending order by the magnitude of the fold change computed between the E18.5 vs E16.5 time points.
(XLSX)

S5 File Summary of Gene Set Enrichment Analysis (GSEA) results. Each row corresponds to a single gene set. Columns A-B contains the MSigDB v5.0 collection and category of each gene set. Column C contains the name of the gene set, represented as a link to the "card" with more information about each gene set at MSigDB. Column D contains the number of genes in the gene set that overlap with the genes in the ranked list. Columns E-H contain the ES (Enrichment Score), NES (Normalized Enrichment Score), nominal *p* value, and FDR *q* value for each gene set. Nominal *p* or FDR *q* values equal to 0 indicate nominal *p* values < 0.001 (i.e., not one shuffled result out of 1000 was more significant than the actual result). The rows are sorted in descending order by NES.
(XLSX)

S6 File Statistical analysis of whole lung gene expression microarray experiment GSE35485. Each row corresponds to a single mouse Entrez Gene. Columns A-E contain the MBNI probeset identifier, links to the Entrez Gene record(s) for the mouse gene and any of its human homolog(s), and the gene symbol and description as obtained from version 17.0.0 of the MBNI mogene10stmmentrezg.db R package. Columns F-G contain the nominal *p* value and FDR *q* value from the moderated one-way ANOVA, and columns H-K contain the signed fold change (computed in linear space), moderated *t* statistic, nominal *p* value, and FDR *q* value for the comparison between the E18.5 and E16.5 time points. All FDR *q* values were computed after excluding any genes that were not expressed above the median value of at least one of all nine samples. Columns L-T contain the log2 (expression) of each gene in all samples, shaded so that blue and red indicate expression values that are at least two standard deviations below and above, respectively, the mean (white) of each row. The rows are sorted in ascending order by one-way ANOVA *p* value.
(XLSX)

S7 File Summary of Gene Set Enrichment Analysis (GSEA) results for PLECs and GSE35485. Each row corresponds to a single gene set. Columns A-B contains the MSigDB v5.0 collection and category of each gene set. Column C contains the name of the gene set, represented as a link to the "card" with more information about each gene set at MSigDB. Column D contains the number of genes in the gene set that overlap with the genes in the ranked list. Columns E-G contain the NES (Normalized Enrichment Score), nominal *p* value, and FDR *q* value for each gene set in the PLECs; columns H-J contain the same values for the whole-lung dataset GSE35485. Nominal *p* or FDR *q* values equal to 0 indicate nominal *p* values < 0.001 (i.e., not one shuffled result out of 1000 was more significant than the actual result). The rows are sorted in descending order by the NES computed for the PLECs, and filtered to show only those gene sets with FDR *q* < 0.25 for the PLECs and FDR *q* ≥ 0.25 for GSE35485.
(XLSX)
